# Retraction: Luminal B tumors are the most frequent molecular subtype in breast cancer of North African women: an immunohistochemical profile study from Morocco

**DOI:** 10.1186/1746-1596-8-127

**Published:** 2013-08-14

**Authors:** Hinde El Fatemi, Sanae Chahbouni, Sofia Jayi, Kaoutar Moumna, My Abdelilah Melhouf, Abdelaziz Bannani, Omar Mesbahi, Afaf Amarti

**Affiliations:** 1 Department of Pathology, Hassan II teaching hospital, Fez, Morocco; 2 Department of Gynecology, Hassan II teaching hospital, Fez, Morocco; 3 Department of Oncology, Hassan II teaching hospital, Fez, Morocco

## Retraction

This article [1] has been retracted by the authors due to extensive overlap with their own previous publication [2] and a number of other sources. In addition, there is duplication of some of the authors previously published results. The authors apologise to all concerned.

## References

[B1] El F Luminal B tumors are the most frequent molecular subtype in breast cancer of North African women: an immunohistochemical profile study from Morocco Diagn Pathol 2012 7 170 10.1186/1746-1596-7-170 23216981 PMC3538531

[B2] Bennis Prevalence of molecular subtypes and prognosis of invasive breast cancer in north-east of Morocco: retrospective study BMC Research Notes 2012 5 436 10.1186/1756-0500-5-436 22889054 PMC3532150

